# 30th Brazilian Society for Virology 2019 Annual Meeting—Cuiabá, Mato Grosso, Brazil

**DOI:** 10.3390/v12050494

**Published:** 2020-04-29

**Authors:** Renata Dezengrini Slhessarenko, Marcelo Adriano Mendes dos Santos, Michele Lunardi, Bruno Moreira Carneiro, Juliana Helena Chavez-Pavoni, Daniel Moura de Aguiar, Ana Claudia Pereira Terças Trettel, Carla Regina Andrighetti, Flávio Guimarães da Fonseca, João Pessoa Araújo Junior, Fabrício Souza Campos, Luciana Barros de Arruda, Jônatas Santos Abrahão, Fernando Rosado Spilki

**Affiliations:** 1 Laboratório de Virologia, Faculdade de Medicina, Universidade Federal de Mato Grosso (UFMT), Cuiabá, MT 78060-900, Brazil; 2 Faculdade de Ciências da Saúde, Universidade do Estado de Mato Grosso (UNEMAT), Caceres, MT 78200-000, Brazil; mardriano@gmail.com; 3 Laboratory of Veterinary Microbiology, Universidade de Cuiabá (UNIC), Cuiabá, MT 78065-900, Brazil; michelelunardi@gmail.com; 4 Instituto de Ciências Exatas e Naturais, Universidade Federal de Rondonópolis (UFR), Rondonópolis, MT 787350-900, Brazil; bruno@ufr.edu.br (B.M.C.); juliana.helena@ufr.edu.br (J.H.C.-P.); 5 Laboratorio de Virologia e Rickettiososes, Faculdade de Medicina Veterinária, Universidade Federal de Mato Grosso (UFMT), Cuiabá, MT 78060-900, Brazil; danmoura@ufmt.br; 6 Nursing Department, Universidade do Estado de Mato Grosso (UNEMAT), Tangará da Serra, MT 78300-000, Brazil; ana.claudia@unemat.br; 7 Collective Health Institute, Universidade Federal de Mato Grosso (UFMT), Cuiabá, MT 78060-900, Brazil; 8 Instituto de Ciências da Saúde, Universidade Federal de Mato Grosso (UFMT), Sinop, MT 78550-728, Brazil; crandrei20@yahoo.com.br; 9 Department of Microbiology, Universidade Federal de Minas Gerais (UFMG), Belo Horizonte, MT 31270-901, Brazil; fdafonseca@icb.ufmg.br (F.G.d.F.); jonatas.abrahao@gmail.com (J.S.A.); 10 Institute of Biotechnology, Universidade Estadual Paulista (UNESP), Botucatu, SP 18607-440, Brazil; joao.pessoa@unesp.br; 11 Bioinformatics and Biotechnology Laboratory, Campus de Gurupi, Universidade Federal do Tocantins (UFT), Gurupi, TO 77410-570, Brazil; camposvet@gmail.com; 12 Laboratório de Genética e Imunologia das Infecções Virais, Depto de Virologia, Instituto de Microbiologia Paulo de Góes, Universidade Federal do Rio de Janeiro (UFRJ), Rio de Janeiro, RJ 21941-902, Brazil; arruda@micro.ufrj.br; 13 One Health Laboratory, Federação de Estabelecimentos de Ensino Superior em Novo Hamburgo (FEEVALE), Novo Hamburgo, RS 93525-075, Brazil

**Keywords:** molecular virology, viral pathogenesis, antivirals, viral epidemiology, viral diseases

## Abstract

The 30th meeting of the Brazilian Society for Virology (SBV) was held, for the first time in its 30 years of existence, in Cuiabá, the capital of Mato Grosso State, Central Western Brazil, a tropical region between the three richest biomes in the world: Amazon Florest, Cerrado and Pantanal. In recent years, the field of virology has been built in the State. The aim of this report is to support participants and virologists to receive the most up-to-date information about the meeting, which occurred from 16 to 19 October 2019. National and international speakers gave SBV the opportunity to learn about their experience on their virology fields, sharing recent scientific findings, compiling conferences, round table presentations and work presentations in oral and poster sessions. The meeting held over 300 attendants, who were also involved on oral and poster presentations, showing a great variety of recent unpublished studies on environmental, basic, animal, human, plant and invertebrate virology. In addition, SBV offered the Helio Gelli Pereira award for the best research studies in each field presented during the meeting. The 30th meeting of SBV was very productive and has also encouraged scientific partnership and collaboration among virologists worldwide.

## 1. Introduction

Virology is a biological research field characterized by rapid advances of concepts, paradigms and understanding of virus biology, epidemiology, pathogenesis, evolution, therapy and control. Viruses have been demonstrated to be the most diverse microorganisms on earth. Human resources and institutions constantly need to update and amplify their resources to go along with this rapid evolution, promoting direct improvements in Brazilian virology education and research institutions. Concerning this, the organizing committee of the 30th Brazilian Society for Virology Meeting provided a coherent scientific program based on national and international high-level selection of researchers to present their recent findings in their fields.

The 30th Brazilian Society for Virology Meeting shared advanced knowledge on important viruses for public health, such as Hepatitis C, Zika, Chikungunya, West Nile and other arboviruses, HIV, HPV, Influenza, oncolytic viruses and those linked to veterinary and agricultural viral diseases. This vast virology approach was explored in order to expand the One Health concept of a global tendency linking human, animal and plant health. The event also allowed students to share their most recent developments, distributed in oral sessions and poster presentations, integrating the academy and giving renowned scientists and new researchers the opportunity to interact in a multi-thematic virological environment.

Therefore, participants experienced in this meeting a unique opportunity to exchange knowledge, share experiences and amplify discussions and extend collaborations with national and international researchers, professors, professionals, postdocs, post-graduate and graduate virology students, reinforcing pre-existing affinities, especially those linked to virologists from Institutions belonging to Mercosul.

It is extremely important to highlight that for the first time the event was held in the state of Mato Grosso, an ancient Brazilian region important as an ecotourism destination related to Pantanal and Amazon, which started to develop as economically noteworthy only in recent years, mostly because of the agriculture field. Virology groups in the State are proportionally few and recent, with low inclusion in graduate courses, research and diagnosis. This scientific area has provided in-depth exploration of the full potential of biodiversity observed in the three biomes located at Mato Grosso State; in such a way, it is expected that these professionals will expand their contributions to the national virology scenario in the near future. The fulfillment of an event of this size in the region allowed local researchers and students of diverse areas within virology to present their skills and form bonds with renowned institutions of other geographic regions so that they can increase and strengthen research in virology fields.

## 2. Historical Perspectives

The Brazilian Society for Virology (SBV) is a non-profit, non-governmental organization that has organized annual scientific meetings since 1986. The Society itself was initiated from discussions between the former Brazilian Microbiology Society president Prof. Golber de Araújo Costa, Dr. Herman Schatzmayr and Dr. Romain Rolland Golgher in Porto Alegre, Rio Grande do Sul State, 1977. In 1978, the most prominent Brazilian virologists at the time met in Florianópolis, Santa Catarina State to discuss the creation of the Society and the Virology Meeting. These first steps led to a strong virologist team being created after 1982. 

Until 2000, the Brazilian virology congress was held in São Lourenço, Minas Gerais State every two years. Since 2001, the meeting was held in different cities every year, including those located in the States of Alagoas (AL), Bahia (BA), Ceará (CE), Distrito Federal (DF), Goiás (GO), Minas Gerais (MG), Pará (PA), Paraná (PR), Rio de Janeiro (RJ), Rio Grande do Sul (RS), Santa Catarina (SC) and São Paulo (SP) States. The latest three SBV events were held in Pirenópolis, GO, Belo Horizonte, MG, and Gramado, RS. In Gramado 2018, the event received 626 attendants, represented mostly by post-graduate students (40%). 

## 3. The scientific Program of the 30th SBV Annual Meeting

The scientific program of the 30th SBV Annual Meeting was based on 38 speakers, 31 Brazilian virology researchers and one from Argentina, four speakers from European institutions—France, Spain and England—and two from USA research groups, distributed over basic, environmental, veterinary, human, plant and invertebrate virology. Speakers represented 8 (21.1%) former SBV members, 22 (57.9%) national and international senior scientists and 8 (21.1%) young researchers distributed in all virology fields that completed their Ph.Ds within less than 10 years, representing the SBV commitment to give an opportunity to recent doctors and research beginners to create solid research lines and interactions with renowned researchers in their fields. All seven conferences and 23 round table speeches were made by men; 14 women presented their research findings in round tables, emphasizing the necessity to improve women’s participation on SBV meetings as speakers. On the other hand, 295 posters were presented by 145 (63%) women and 85 (37%) by men; 25 (65.8%) oral presentations were made by women and 13 (34.2%) by men; 6 (50%), although awarded oral presentations (*n* = 12), were made 50% by women, 50% by men. These statistics show that we must improve and recognize women’s participation in the most relevant activities in future meetings since they represent the majority of participants, posters and oral presenters nowadays in Brazilian virology fields.

Speakers were distributed over seven main conferences and 12 round tables, including four presentations of the most recent subjects on the fields of antivirals, plant virus diversity and evolution, virology of plants and invertebrates, virus–cell interactions, emerging viruses, oncolytic immunotherapy, viral structure and pathogenesis, viral global spread, small animal viruses, omics virology and environmental virology. 

The 30th SBV Annual Meeting also hosted eight sections of students’ oral presentations, two sharing basic, human and veterinary research studies, one of plant and invertebrate and finally, one of environmental virology. All of these eight sections of oral presentations, in addition to poster presentations by virology field, applied for the Helio Gelli Pereira (HGP) award, which this year granted a free publication fee on Viruses Journal for the two general winners and a fee deduction for each field first position in oral and poster presentation.

More information about the event may be found on the meeting website, http://congressovirologia.com.br/2019/ [1] and on the society website https://sbv.org.br/sbv/ [2].

### 3.1. Attendants at the Meeting

**Figure 1:**
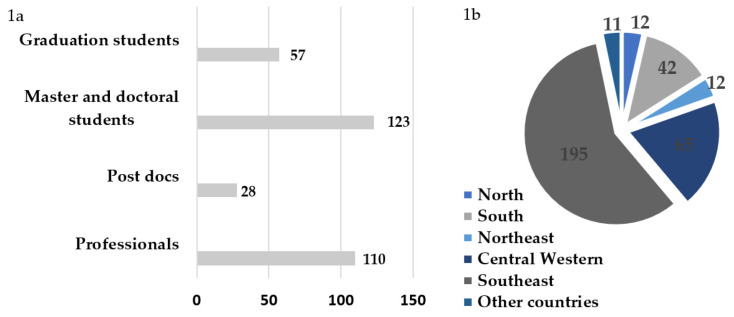
Participants of the 30th Brazilian Society for Virology Congress and 14th Mercosul Virology Meeting by category (**a**) and their respective institution locations in each country region (**b**).

The 30th SBV Annual Meeting attendees were characterized by professionals (33%), post-doc students (8%), Master and Ph.D. students (36%) and graduate students (23%), represented by participants from Southeast (58%), Central Western (19%), South (12%), North (4%), Northeast (4%) Brazil and international participants from Mercosul countries (3%), comprising 318 participants (Figure 1b). About 60% of all the meeting participants were women and 40% were men.

### 3.2. Scientific Program

For four days, the 2019 SBV occurred in Centro de Eventos do Pantanal, a strategic area located in the capital of Mato Grosso (MT) State, Cuiabá. The event was divided amongst the facilities of this modern and sustainable building with simultaneous conferences and round tables (Table 1).

### 3.3. Conference Speakers and Presentations

Dr. Elliot Watanabe Kitajima is a plant virologist from Escola Superior de Agricultura Luiz de Queiroz, Universidade de São Paulo (USP), Brazil. Over his career, he has contributed to the discovery and characterization of several plant viruses, resulting in more than 400 research articles, 12 books and 31 book chapters. Due to his impressive contributions, a viral family was named in his honor as the *Kitaviridae* family. His main opening conference was based on plant viruses transmitted by *Brevipalpus saga*.

Dr. Mauricio Lacerda Nogueira is an infectologist from São José do Rio Preto School of Medicine from (FAMERP), Brazil, and a former president of SBV (2017-2019). Since his Ph.D. in the National Institute of Allergy and Infectious Diseases from the USA, Dr. Nogueira has developed an impressive work in arbovirology, contributing to several studies during the Zika virus introduction and dispersion in Brazil. In our meeting, he shared his experiences with the conference “Interplay between dengue and Zika: what an endemic area can teach us”. 

Dr. Amauri Alcindo Alfieri from Universidade de Londrina (UEL), Brazil is an animal virologist. His research over the years brought relevant contributions in the diagnosis and molecular epidemiology of enteric viral agents classified in diverse viral families and affecting swine and cattle. His conference focused on the description of important veterinary enteropathogenic viruses naturally occurring in Southern Brazil. 

Dr. João Trindade Marques has developed important studies in immunology and virology at Universidade Federal de Minas Gerais (UFMG), Brazil. His conference entitled viral interaction, iRNA and arboviruses focused in his recent work showing insect-specific viruses’ diversity through iRNA and virus–host interplay, extending our knowledge that these viruses may either enhance or reduce arbovirus replication in competent vectors.

Dr. Luiz Carlos Junior Alcântara is a researcher at Fundação Oswaldo Cruz (Fiocruz) from Bahia and a bioinformatics collaborator at UFMG, Brazil. Recently, Dr. Alcantara has traveled the whole country sequencing arbovirus samples from Central public health laboratories by a real-time MiniIon platform named the ZIBRA project. This conference showed partial results obtained on arbovirus real-time surveillance in Brazil. 

Dr. Valerian Dolja, a researcher at Oregon State University from the USA, showed his impressive studies on virus evolution in his presentation “Origin of viruses: primordial replicators recruiting capsids from hosts”. His group publications have changed the way that the International Committee on Virus Taxonomy (ICTV) and virologists around the world currently classify viruses.

Dr. Glenn C. Randal from University of Chicago, USA, is a human virologist developing research on Dengue and hepatitis C pathogenesis. In our meeting, Dr. Randal shared his studies on Hepatitis C virus–host interactions.

### 3.4. Round Tables

Round table 1 comprised environmental virology and had Dr. Gislaine Fongaro (Universidade Federal de Santa Catarina, UFSC) as Chairperson. Dr. David Rodriguez-Lazaro (University of Burgos, Spain) contributed to the field with “Hepatitis E virus: an emerging foodborne pathogen?”, whereas Dr. Marcelle Figueira Marques da Silva (Universidade Federal de Goiás, UFG) shared her knowledge about rotavirus and vaccination importance. Finally, Dr. Maria Tereza Pepe Razzolini (USP) spoke about virological risk management.

Round table 2 on basic virology field had Dr. José Luiz Proença Módena (Universidade de Campinas, UNICAMP) as chairperson and presented recent metagenomic data from Dr. Thiago Moreno Lopes e Souza (Fiocruz, Rio de Janeiro), entitled “Omics to reinforce laboratory-based surveillance”. Dr. Gustavo Bueno Gregoracci (Universidade Federal de São Paulo, UNIFESP) presented his work on “Viral metagenomic diversity in coastal and marine environments”, and Dr. William Marciel de Souza (UNICAMP) finished the section with his presentation “virus hunting in the genomic era”.

Invertebrate virology comprised round table 3, conducted by Dr. Daniel Mendes Pereira Ardisson de Araújo (Universidade Federal de Santa Maria, UFSM), who also spoke about Iflavirus–host interaction. This section also included Dr. Bergmann Morais Ribeiro (Universidae de Brasilia, UNB) presentation on Molecular biology of *Chrysodeixis includens* viruses, and Dr. Stephane Blanc’s (Institut national de la recherche agronomique, INRA, France) speech entitled “Nanovirus turns its aphid vectors into walking-dead”.

Small animal viruses were the theme of round table 4, with Dr. Mathias Martins (Universidade do Oeste Catarinense, UNOESC) as Chairperson. Dr. Alice Fernandes Alfieri (UEL) shared her findings on feline morbillivirus perspectives in Brazil, whereas Dr. Pablo Sebastian Britto de Oliveira (UFSM) spoke about “Epidemiological, clinical-pathological and genetic features of canine parvovirus (CPV-2) in dogs in Southern Brazil”. Dr. Abelardo Silva Junior (Universidade Federal de Viçosa, UFV) closed this section speaking about drug and gene therapy for canine morbillivirus.

Round table 5 was dedicated to viral global spread. Dr. Isabel M.V.G. Carvalho Mello (Instituto BUTANTAN) conducted, Dr. Soniza Vieira Alves Leon (Universidade do Rio de Janeiro, UniRio) gave a presentation about Chikungunya virus and neurological spectrum disorders, Dr. Elba Regina Sampaio de Lemos (Fiocruz, Rio de Janeiro) gave a speech on “Hantavirus and arenavirus in Brazil: an unpredictable and latent human health threat” and Dr. Eurico de Arruda Neto (USP), a former SBV president, gave a speech on “Viruses that we carry around: puzzles of human lymphoid tissue virome”.

Viral structure and pathogenesis were the theme of round table 6, conducted by Dr. Rafael Elias (Laboratorio Nacional de Biosciências, LNBio). He introduced Dr. Felix A. Rey’s (Instituto Pasteur, France) speech about “Structure-guided reverse vaccinology approaches to protect against enveloped viruses”, Dr. André Marco de Oliveira Gomes (Universidade Federal do Rio de Janeiro, UFRJ) gave a presentation about “arbovirus envelope lipid composition and virus cell-interactions”, and finally, Dr. Andre S. Godoy (USP) shared his studies on “Targeting flavivirus non-structural proteins for drug discovery”.

Emerging viruses were discussed on round table 7, which had Dr. Abelardo Silva Junior (UFV) as chairperson. Dr. Helena Lage Ferreira (USP) presented her work on pathogenesis and transmission of virulent newcastle disease viruses, whereas Dr. Érica Azevedo Costa (UFMG) reported the recent “Detection of West Nile virus in equids in Brazil: challenges and perspectives”. Dr. Amauri Alcindo Alfieri (UEL) closed this table by presenting his studies with Senecavirus in Southern Brazil.

Round table 8’s subject was oncolytic immunotherapy. Dra. Paula Rahal (Universidade do Estado de São Paulo, UNESP) conducted the presentations by Dr. Marzia Puccioni Sohler (UFRJ) with the theme “HTLV-1 infection: a neglected health problem in Brazil”, Dr. Luís Carlos de Souza Ferreira (USP) spoke about “Active immunotherapy: a novel therapeutic concept to control HPV induced-tumors,” and Dr. Martin Hernan Bonamino (Instituto Nacional do Cãncer, INCA) showed his studies with transposon-based cancer immuno-gene therapy.

Plant and invertebrate virology round table 9 had as chairperson Dr. Poliane Alfenas Zerbini (UFV). Tospovirus–host interaction and Polerovirus–host interaction were presented by Dr. Renato de Oliveira Resende (UnB) and Dr. Maite Vaslin de Freitas Silva (UFRJ), respectively. Dr. Victoria Alfonso (Instituto Nacional de Tecnología Agropecuaria, Argentina) spoke about her group studies on “Baculovirus ACMNPV: impact on the development of biotechnological tools”.

Round table 10 debated virus–cell interactions. The chairperson was Iranaia Assunção-Miranda, (UFRJ), Dr. Glenn C Randall (University of Chicago, USA) presented a speech about flavivirus modulation of cellular lipids metabolism. Then, Dr. Luciana Jesus da Costa (UFRJ) presented her recent studies on “Antiretroviral drugs: new approaches, new targets”, followed by Dr. Renato Santana de Aguiar’s (UFMG) presentation about common cellular pathways and gene regulation expression mechanisms modulated by encephalitis causing arboviruses (Zika, Chikungunya, Oropouche and Mayaro). 

Antivirals were the subject of round table 11, chaired by Dr. Renato Santana de Aguiar (UFMG).

Dr. Luciana Jesus da Costa (UFRJ) spoke about “New designs, new approaches, new targets, new viruses”, and Dr. Bruno Moreira Carneiro (Universidade Federal de Rondonópolis, UFR) about a novel approach to HCV therapy. Dr. Carla Regina Andrighetti (Universidade Federal de Mato Grosso, UFMT) spoke about her studies on antiviral activity of natural products from Mato Grosso State. This section was closed by Dr. Eduardo J. M. Nascimento (TAKEDA Vaccine Unit, England), who brought a presentation for understanding the immune response to dengue infection and vaccination.

The last round table of the event, chaired by Dr. Daniel M.P. Ardisson-Araujo (UFSM), focused on plant virus diversity and evolution. Dr. Stephane Blanc (INRA, France) presented his interesting findings on new conceptual frameworks that are required to understand the biology of multipartite viruses, Dr. Valerian Dolja (Oregon State University, USA) spoke about origins and evolution of the global RNA virome and Dr. Francisco Murilo Zerbini Junior (UFV) concluded the table speaking about “Evolutionary dynamics of bipartite begomoviruses: one genome, two histories”.

### 3.5. Poster and Oral Presentations and the Helio Gelli Pereira Award

Poster and oral submissions represented 292 unpublished works, distributed as basic virology (44, 15%), human and public health virology (129, 44%), environmental virology (30, 10%), veterinary virology (49, 17%), plant and invertebrate virology (28, 10%) and immunobiologicals (13, 4%). From these, 39 studies were selected for oral presentations, distributed between veterinary virology (10), basic virology (10), human virology (9), environmental virology (5) and plant and invertebrate virology (5). Posters and oral presentations awarded by the virology field are presented in Table 2.

The Helio Gelli Pereira award was granted to oral presentations of postgraduate student Severino Jefferson Ribeiro da Silva from Instituto Aggeu Magalhaes, Fiocruz, Recife, PE, whose work was entitled “Development and validation of a reverse transcription Loop-mediated isothermal amplification (LAMP) for rapid detection of ZIKV in mosquito samples from Brazil” and to undergraduate student Roberta Corrêa Cahù from Universidade de Brasília, DF, with her work “Production of YFV and HIV virus like particles (VLPs) using baculovirus expression system and insect cells” [15]. The award was supported by SBV, American Society for Microbiology (ASM) and Viruses. ASM granted a 1-year membership to ASM and full discount for publication of the awardees, if the manuscript was accepted by referees and editors, respectively. One of the works was recently published in Viruses [16].

## 4. Concluding Remarks

The 30^th^ Brazilian Society for Virology Meeting provided a unique opportunity for scientists, undergraduate and postgraduate students, professionals and principal investigators to share information and exchange experiences in virology with a broad perspective. The meeting explored recent research findings in all the main fields of virology, reinforcing the importance of this knowledge area for modern science. The fact that this meeting was held for the first time in Cuiabá, South-Central Mato Grosso strengthens the virology field in the State through a network of international and national collaboration. Therefore, SBV also invests in recent researchers’ consolidation and enhances its purpose, which is to promote the exchange of information and to stimulate discussion and collaboration among virologists bringing the virology field as a key point in science and development. Consolidation of virology in Brazil strongly reinforces rapid and efficient responses to the emergence and re-emergence of public, animal and agriculture health-important viruses.

## Figures and Tables

**Table 1:** Scientific programming schedule of the 30th Brazilian Society for Virology Congress and 14th Mercosul Virology Meeting.

**Hour**	**Flowers Auditorium**	**Trees Auditorium**
**Wednesday, 16th**		
15:00–18:00	PRE-CONGRESS ACTIVITY # 1 ASM Workshop on science communication and outreach	-
18:30 –20:00	OPENING SESSION Conference 1 Plant viruses transmitted by Brevipalpus saga	-
20:00–22:00	Confraternization cocktail	
**Thursday, 17th**		
9:00–10:15	Conference 2 Interplay between Dengue and Zika: what an endemic area can teach us	Oral presentations session 1 Veterinary virology 1
10:15–10:45	Coffee break and visit to exhibits	
10:45–12:00	Conference 3 Veterinary important enterophatogenic viruses	Oral presentations session 2 Basic virology 1
12:00–13:30	Lunch break	
13:30–15:15	Round table 1 Environmental virology	Round table 2 Omics virology
15:15–15:45	Coffee break and visit to exhibits	
15:45–17:15	Round table 3 Invertebrate virology	Oral presentations session 3 Human virology 1
17:15–18:30	Conference 4 Viral interaction, iRNA and arboviruses	-
18:30–20:00	Poster Section 1	
**Friday, 18th**		
9:00–10:15	Round table 4 Small animal viruses	Round table 5 Viral global spread
10:15–10:45	Coffee break and visit to exibits	
10:45–12:00	Conference 5 Arboviruses real time surveillance	Round table 6 Viral structure and pathogenesis
12:00–14:00	Lunch break	
14:00–15:30	Oral presentations session 4 Basic virology 2	Oral presentations session 5 Environmental virology
15:30–16:00	Coffee break and visit to exibits	
16:00–17:15	Conference 6 Origin of viruses: primordial replicators recruiting capsids from hosts	Round table 7 Emerging viruses
17:15–19:00	Round table 8 Oncolytic immunotherapy	Round table 9 Plant and invertebrate virology
19:00–20:30	Poster Section 2	
**Saturday, 19th**		
9:00–10:15	Helio Gelli Pereira award presentations	Oral presentations session 6 Veterinary virology 2
10:15–10:45	Coffee break and visit to exibits	
10:45–12:00	Round table 10 Virus cell interactions	Oral presentations session 7 Human virology 2
12:00–14:00	Lunch break	
14:00–15:15	Round table 11 antivirals	Oral presentations session 8 Plant and invertebrate virology
15:15–15:45	Coffee break and visit to exibits	
15:45–17:30	Conference 7 Hepatitis C virus–host interactions	Round table 12 Plant virus diversity and evolution
17:30–18:00	Helio Gelli Pereira award	-
18:00–19:00	SBV general assembly	-
21:30	Confraternization	

**Table 2:** Poster and oral presentations awarded at the 30th Brazilian Society for Virology Congress and 14th Mercosul Virology Meeting.

**Presentation**	**First Author-Filiation Institution**	**Title**
**Veterinary Virology**		
Poster 264	André Ferreira Hennigen UFRGS	Virome of the nasal cavity of swine prior to slaughter [3]
Oral	Gabriela Molinari Darold UNIC	Molecular investigation of the presence of feline paramyxovirus RNA in kidneys of domestic cats from Cuiabá, Mato Grosso [4]
**Basic Virology**		
Poster 159	Cristina Santos da Costa USP Ribeirão Preto	The host protein AP1 is relevant to HIV-1 NEF antagonism against serinc 5 [5]
Oral	Daniel Augusto de Toledo Teixeira-Unicamp	The antibody production and innate immune response by B cells are essential for restriction of Oropouche virus prime-infection [6]
**Human Virology**		
Poster 219	Marcela Helena Gonçalves Pereira-UFMG	Study of immunoregulatory mechanisms mediated by regulatory T cell during Dengue infection in humans [7]
Oral	Marcilio Jorge Fumagalli USP Ribeirão Preto	Previous CHIKV exposure induces partial cross-protection against secondary MAYV infection in mice [8]
	Bruna Lais Santos de Jesus–USP Ribeirão Preto	Infection of lymph nodes by respiratory syncytial virus [9]
**Environmental Virology**		
Poster 001	Lorhan Lima Leal UFV	A new dsRNA mycovirus infecting the phytopathogenic fungi *Mycosphaerella fragariae* [10]
Oral	Doris Sobral Marques Souza-UFSC	Influence of faecal contamination from the Camboriu river on the microbiological quality of water in a bivalve shellfish production area [11]
**Plant and Invertebrate Virology**		
Poster 252	Fabricio da Silva Morgado-UnB	Ultrastructural studies of the cotesia flavipes ovaries and its endosymbiotic polydnavirus [12]
Oral	Luca Cestari-Embrapa	An in-silico approach to validate the capsid architecture of new putative icosahedral viruses: geminiviridae as case study [13]
**Immunobiologicals**		
Poster 246	Monica Josiane Rodrigues de Jesus–USP São Paulo	Nanomultilamellar lipid vesicles potentialize the IgG antibody responses against Zika virus NS1 protein [14]
